# Obstetrics and Gynecology

**Published:** 1893-09

**Authors:** 


### OBSTETRICS AND GYNECOLOGY.

EDITED BY DR. VIRGIL O. HARDON.

### ALBUMINURIA IN RELATION TO PARTURITION.

Aufrecht (*Centralb. f. klin. Med.*, June 3d, 1893) records some investigations in which the urine was examined before and after labor, all possible sources of contamination being avoided. Of thirty-two cases none had albumin before labor or just after the commencement of pains, but in eighteen albumin, varying in quantity from one to two to one-fourth pro mille, was found after labor. In all these cases the albumin disappeared in twenty-four hours. Epithelium, but no casts were present. It is concluded that labor, or rather the pains, cause the albuminuria by producing some stagnation in the nervous system, including the renal veins. Thus the urine should be examined before labor, and if albumin is present the case should be watched, as an increase is likely to occur during the labor. If eclampsia appears at the beginning of or during the pains, the labor should be hurried on as much as possible, as, according to the author's experience, renal disease with albuminuria is without exception the cause of eclampsia. The function of the kidneys is further compromised by long-lasting pains, and the danger to the mother increased. Where artificial delivery cannot yet be effected, chloral is most suitable. Again, albuminuria and cylindruria are separate processes, the latter having nothing to do with the transudation of albumin through the renal vessels. Casts are the product of inflammatory irritation of the renal epithelium.—*British Medical Journal.*

### CLINICAL STUDY OF CERVICAL DYSMENORRHEA.

Hanfield-Jones (*Brit. Med. Jour.*) criticizes the attempt of authors to refer all cases of uterine dysmenorrhea to a single cause, and cites a number of cases from his own practice to show how many different factors may be active. Among these are the neuralgic, inflammatory and spasmodic types. The writer believes that rhythmical uterine contractions occur at the beginning of menstruation, which, with simultaneous inhibition of the sphincter fibers, cause dilatation of the cervical canal. Under normal conditions these phenomena are unaccompanied by pain. Any pathological condition which delays the process of dilatation is attended with painful menstruation, such as displacement of the uterus, muscular spasm at the os internum, fibroid thickening at the same point, and hyperesthesia of the nerve-endings resulting from endometritis. Practically, when cervical endometritis is present, hot douches, leeching, and applications of carbolic acid or iodized phenol are indicated, but when no such disease can be found dilatation offers the most certain means of relief.—*Am. Jour. Med. Sciences.*

---

### THE RELATION OF PELVIC PERITONITIS TO ECTOPIC PREGNANCY.

Martin (*Berliner klinische Wochenschrift, 1893, No. 22*), after giving the opinions of various authors on the relation of pelvic peritonitis to ectopic pregnancy, records that, in his own opinion, in the majority of cases the peritonitis acts more as a rapid consequence of the ectopic insertion of the ovum than as a cause of this abnormality. The growing ovum works unmistakably as a center of inflammation, causing not unimportant changes in the immediate and distant neighborhood, such as adhesions and other changes found after pelvic peritonitis from other causes. Also against the theory of tubal disease offering a cause for ectopic gestation, he argues that the impregnated ovum requires a healthy situation in which to grow, and thinks it very uncertain that it is possible for it to develop on the diseased mucous membrane of the uterus, much less in an unhealthy tube. The mucous membrane may have previously been diseased, but must be healthy at the time of the impregnation and attachment of the ovum.

The author then describes a case on which laparotomy was performed for tubal pregnancy. The tumor was the size of a pigeon's egg. The omentum and the peritoneum, parietal and visceral were engorged with blood. The ovum, which was perhaps four weeks advanced in gestation, was situated between the fimbriated extremity and the ovary. The ovary was considerably enlarged and was partially covered by a rough covering. Its surface was considerably congested, and had remaining a single follicular elevation.—*Am. Jour. Med. Sciences.*

---

### MEMBRANOUS DYSMENORRHEA.

Dr. Lohlien (*Gyn. Tagesfragen, Heft II., 1891*) reviews the subject at length and confesses to changes of opinion on several points. The name is a misnomer, as membranes may be passed at the menstrual period without pain, hence the French *dysmenorrhée membraneuse sans dysmenorrhie.* He recognizes different degrees of the affection, and would designate the severer forms as "exfoliative endometritis." It has not as yet been definitely ascertained why this affection should occur in the most varied diseases of the uterus and its adnexa. In the milder variety the superficial layers of the membranes are cast off by the occurrence of small hemorrhages in the superficial strata of the uterine mucous membranes. But a different process obtains in the pronounced variety. There a true interstitial endometritis occurs involving the glands. The author has seen it frequently follow a puerperium complicated by inflammatory conditions. In one-third of his cases (twenty-seven) he could trace no etiological factor. Different from the experience of other observers, six of his patients bore children after the appearance of the membranous casts. He had seen only one case which could have been said to have undergone complete cure, and this result could not have been attributed to the local treatment employed. But scraping the uterus, and following it by intra-uterine injections, has been attended with a cessation of the affection for several months.

### CAUSATION OF THE DISEASES OF WOMEN.

If proper attention were given to growing girls, especially about the time of puberty, and a more normal development of the sexual organs secured, if gonorrhea were more vigorously treated, and if the subjects of that disease were kept under observation until all abnormal discharges were arrested, and proper instructions concerning the abstention from sexual intercourse were given; if antiseptic midwifery were faithfully and efficiently practiced; if lacerations of the cervix and perineum were early repaired; and if full instructions concerning the ill effects of constipation, improper dress, and erroneous habits of living were given, the prevalence of the diseases peculiar to women would be very greatly restricted. I believe that this is to be the next great advance in diseases of women. Gynecologists must bring home to the general practitioner the fact that the diseases of women are largely preventable, and make him feel his responsibilities, both as to their production after present methods of practice and as to the possibilities of their prevention after improved methods. When the family physician realizes that it lies within his power very largely to prevent disease among the women of the families committed to his care, his sense of moral obligation will spur him on to do his full duty in this matter. When that day comes the universal prevalence of disease among women will cease to be a reproach to preventive medicine.—*C. P. Noble, Int. Med. Mag.*

---

### HOW TO ASCERTAIN A TWIN PREGNANCY.

It is best done by abdominal palpation and auscultation.

In twin conception, on uncovering the woman's abdomen, one can at once notice the considerable dimensions of the uterus, the irregularity of its shape, a depression, even a sulcus, crossing obliquely the abdominal walls.

This sulcus is always present when the two fetuses are lying obliquely above the other, as generally happens. But it does not exist when the fetuses are in front of the other.

By abdominal palpation, the diagnosis is easy in the first instance, but difficult in the other instance.

At any rate, palpation at once reveals the great volume of the uterus and its irregular shape. But its tension, on account of a greater amount of amniotic fluid, renders the diagnosis more difficult, as the fetal parts are not so well defined.

By palpation it can be ascertained that in twin pregnancy the large fetal tumors are double; for instance, one head can be found near the superior strait, the other at the fundus of the uterus; and one back, in an oblique and inferior direction.

In other instances, there may be found two breeches, two backs, and only one head in one of the iliac fossæ; the other head, being concealed in the excavation, can be found by the vaginal touch only. This is a very delicate point in obstetric diagnosis.

*Auscultation* will generally much assist in ascertaining the beat of two hearts at different points.—*Boisliniere, American Gynecological Journal.*

---

### VULVO-VAGINITIS IN CHILDREN.

Williams, of Baltimore (*Maryland Medical Journal*, June 11, 1892), cites eight cases of leucorrhea in little girls seen in the dispensary practice at Johns Hopkins Hospital. The children varied in age from two to eight years. The vaginal discharge had been present from one day to one year. Five of the cases were examined as to the presence of microörganisms; of these four contained what appeared to be gonococci. He believes that most cases are infectious, and in all probability of gonorrheal origin. The most frequent mode of infection he believes to be that indirectly from the mother, or some other member of the family, by means of the general use of the same toilet articles, or by the children playing with each other's genitals. Williams insists upon the most scrupulous cleanliness in the treatment of his cases to prevent reinfection. The treatment comprises the use of iodoform suppositories and the application of silver nitrate solution, thirty grains to the ounce. The nitrate of silver is best applied to the vagina twice a week on a tuft of cotton twisted upon the end of a probe. The genitals should be frequently cleansed with castile soap and warm water and then dusted with boracic acid.

